# Supplementary material for: Differences in the Residual Behavior of a Bumetrizole-Type Ultraviolet Light Absorber during the Degradation of Various Polymers
Source: Polymers (Basel). 2024 Jan 21;16(2):293. doi: 10.3390/polym16020293 (PMC10819654; doi:10.3390/polym16020293)
Supplement: Supplementary file 1 [file polymers-16-00293-s001.zip › polymers-2818573-supplementary.pdf]

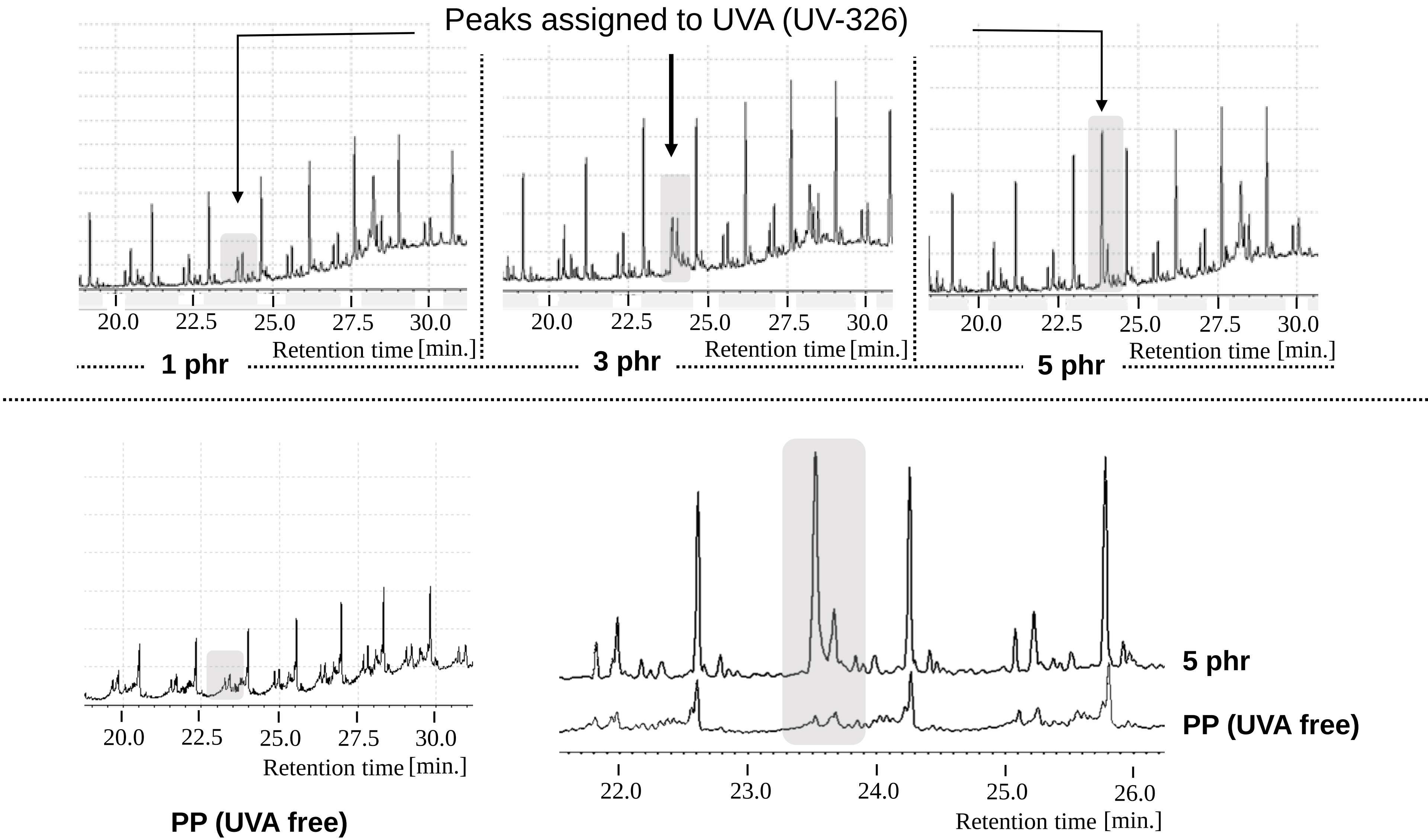

Figure S1 Pyrolysis gas chromatography (Py-GC/MS) charts of PP samples containing various UVA concentration.

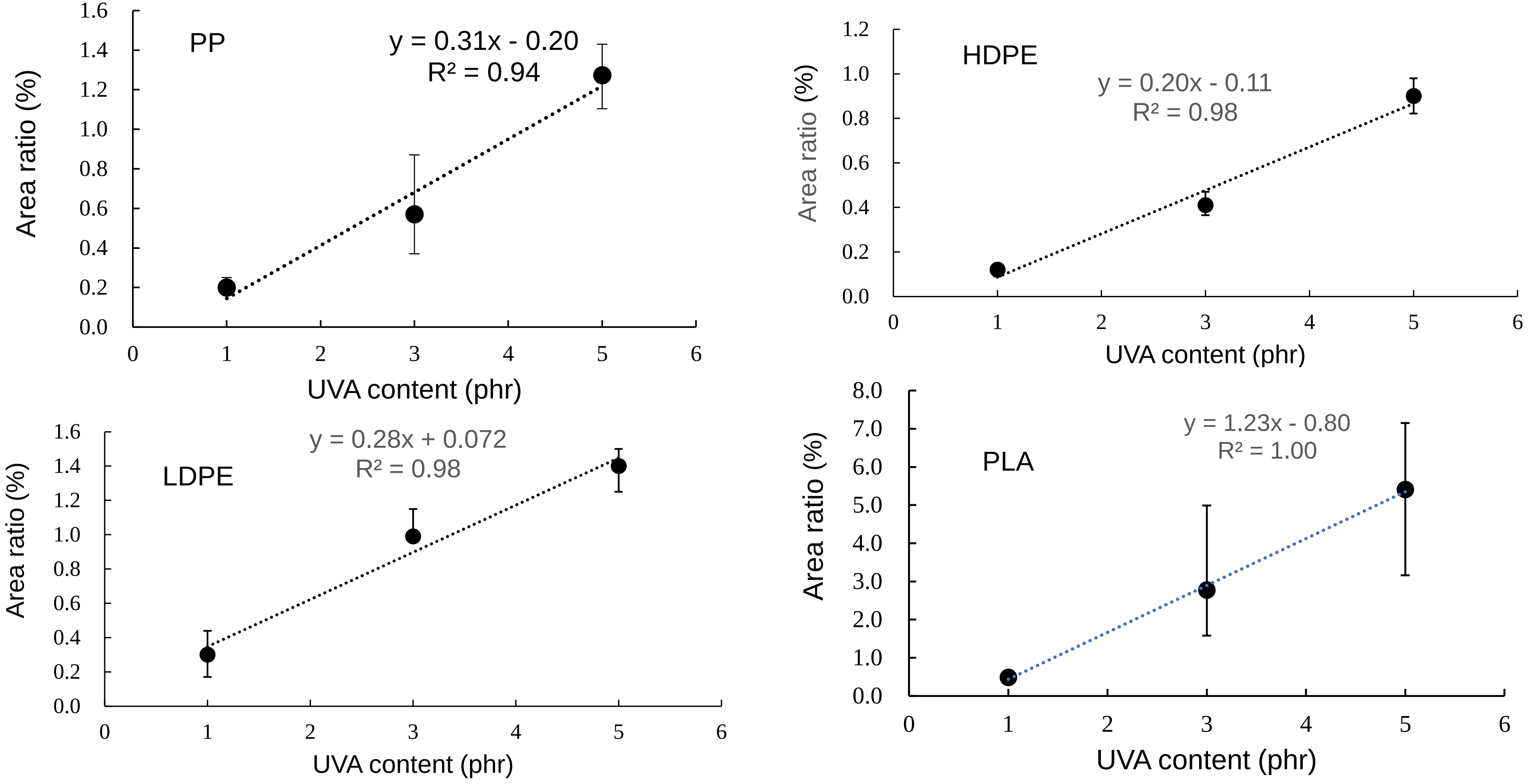

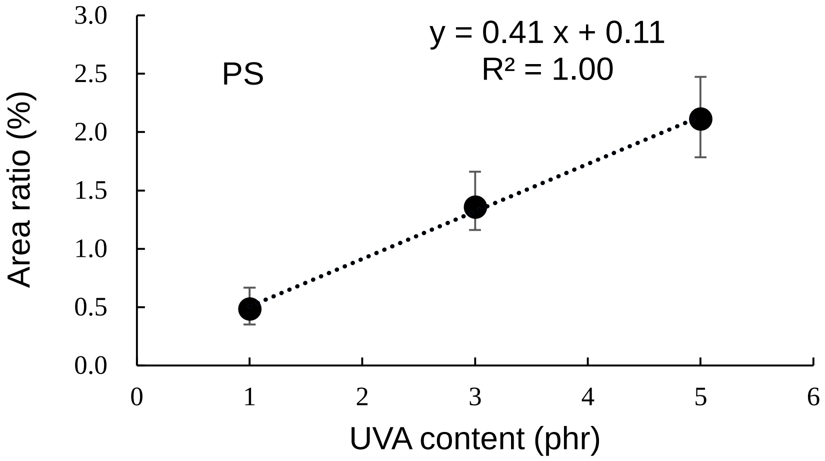

Figure S2 Relationship between UVA concentration in various polymers and area ratio obtained from pyrolysis gas chromatography (Py-GC/MS) measurements: Number of measurements = 3 times.

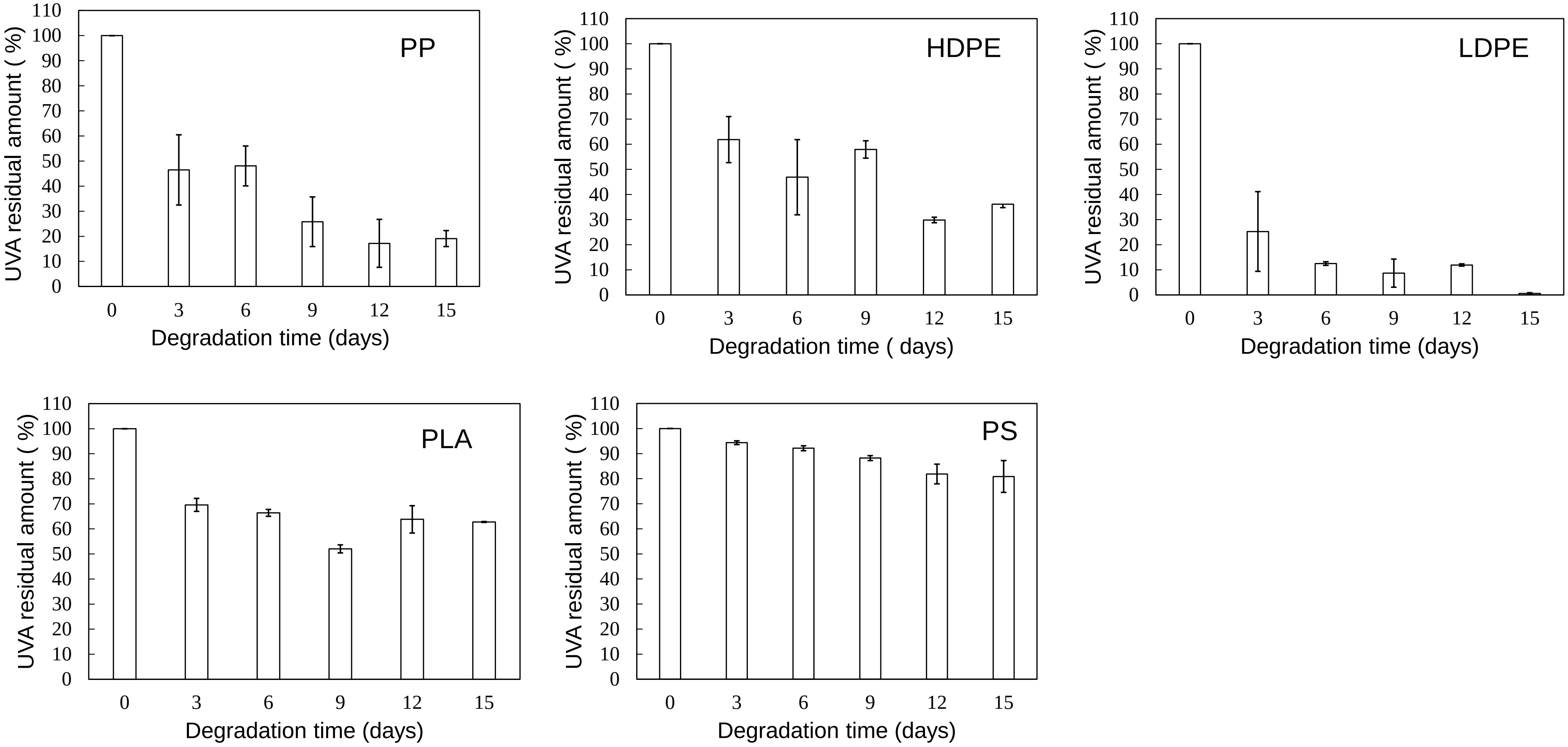

Figure S3 Degradation time dependence of UVA residual amount in various polymer samples using sulfate ion radical in pure water.

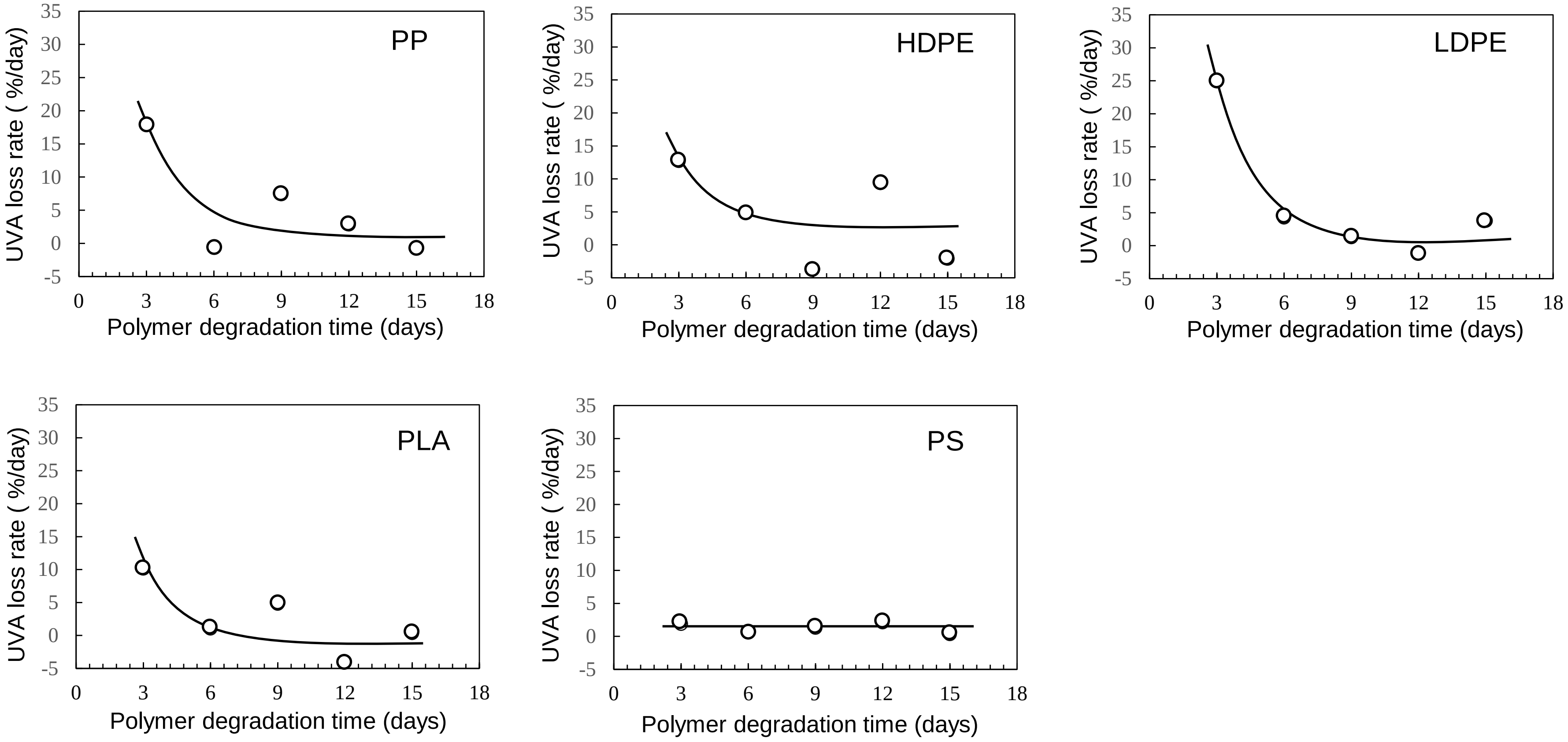

Figure S4 Degradation time dependence of UVA loss rates in various polymer samples using sulfate ion radical in pure water.

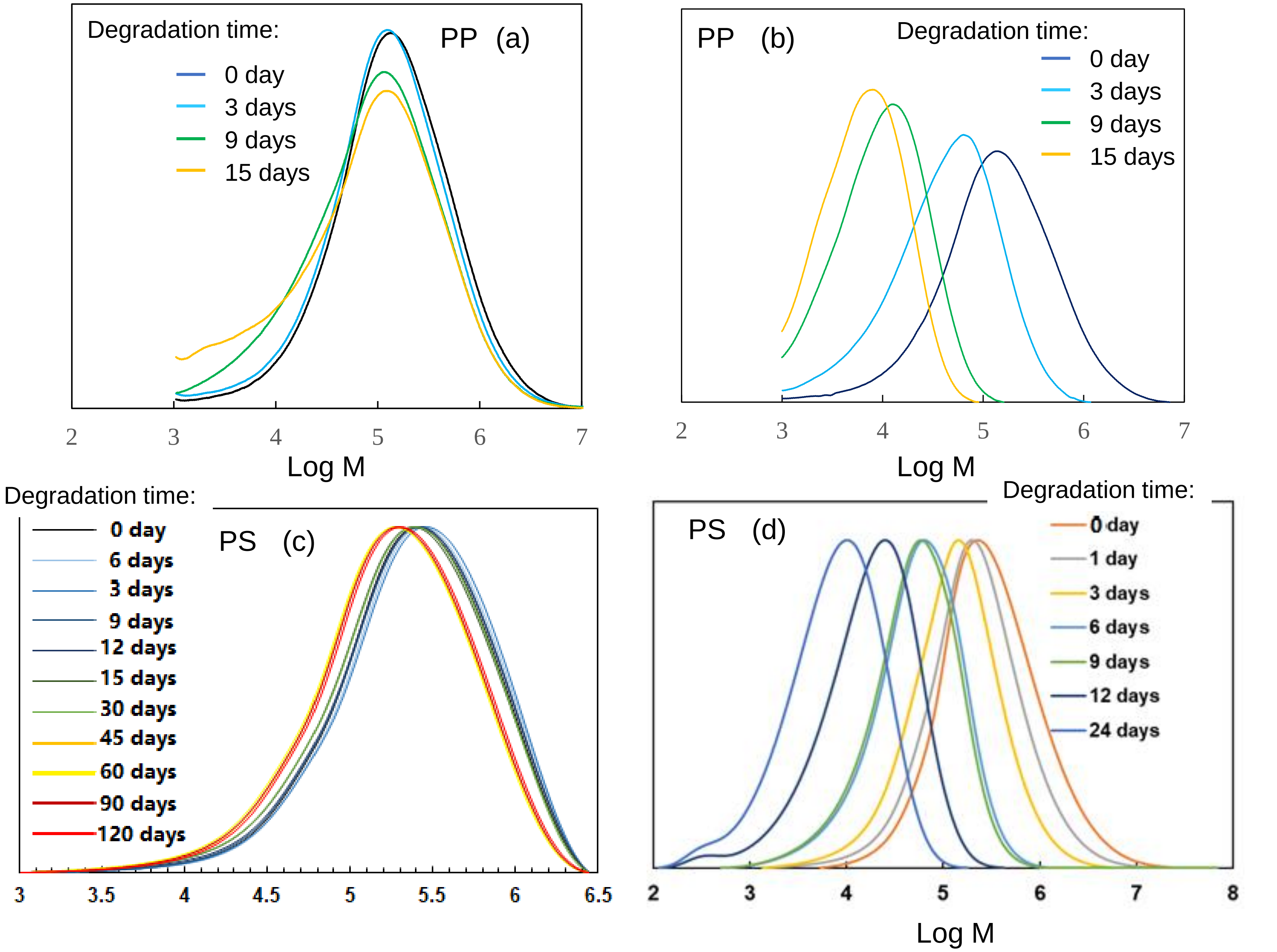

Figure S5 Molecular weight curve changes of PP and PS samples during degradation time : (a) by sulfate ion radical in pure water. (b) by sulfate ion radical in seawater (EDM). (c) by sulfate ion radical in pure water. (d) by sulfate ion radical in seawater (EDM).

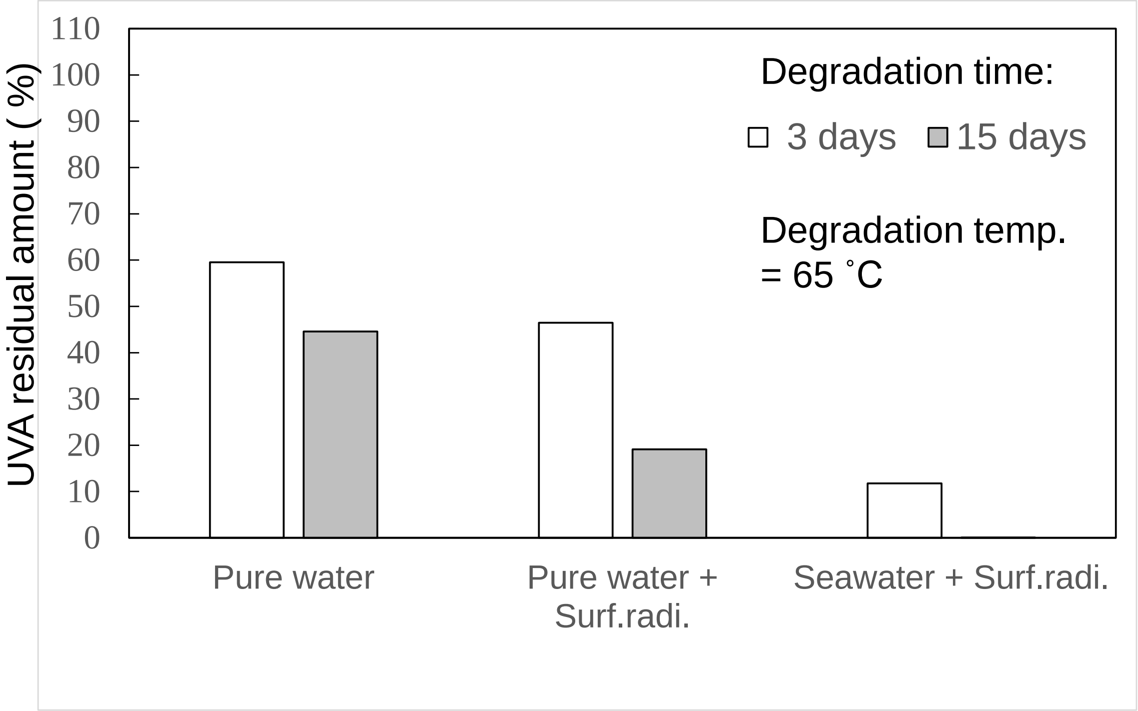

Figure S6 Comparisons of degradation time dependence of UVA residual amounts in PP samples in pure water, using sulfate ion radicals in pure water, and using sulfate ion radicals in seawater (EDM): All degradation temperatures = 65 ˚C;

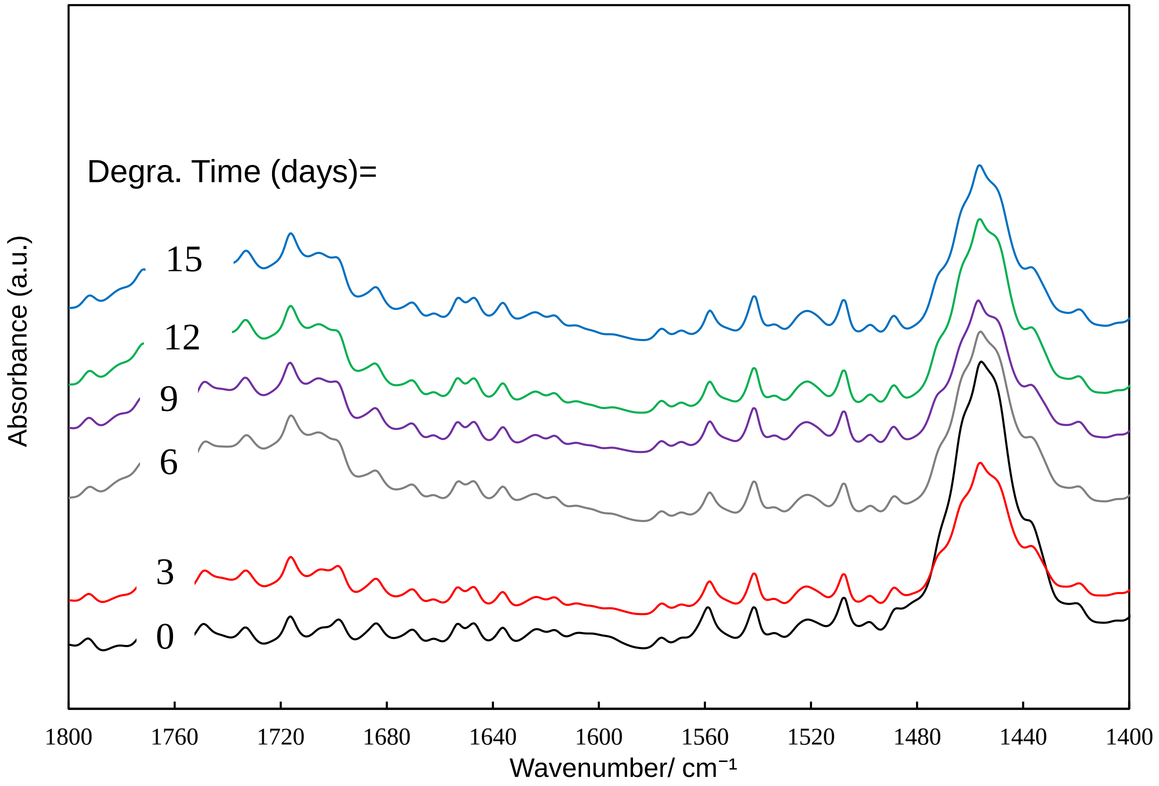

Figure S7 IR spectra changes of PP containing 5-phr UVA film through EDM degradation.

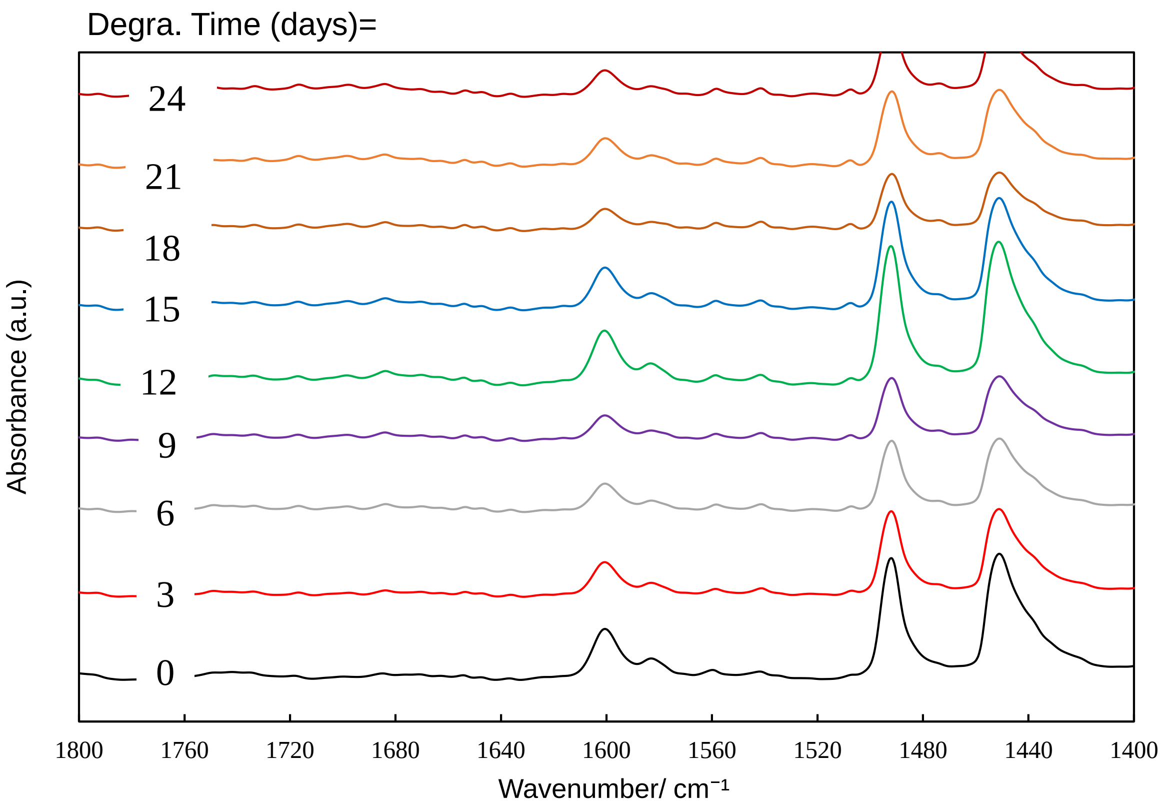

Figure S8 R spectra changes of PS containing 10-phr UVA film through EDM degradation.
